# Rise of Deep Learning Clinical Applications and Challenges in Omics Data: A Systematic Review

**DOI:** 10.3390/diagnostics13040664

**Published:** 2023-02-10

**Authors:** Mazin Abed Mohammed, Karrar Hameed Abdulkareem, Ahmed M. Dinar, Begonya Garcia Zapirain

**Affiliations:** 1 College of Computer Science and Information Technology, University of Anbar, Anbar 31001, Iraq; 2 eVIDA Lab, University of Deusto, 48007 Bilbao, Spain; 3 College of Agriculture, Al-Muthanna University, Samawah 66001, Iraq; 4 College of Engineering, University of Warith Al-Anbiyaa, Karbala 56001, Iraq; 5 Computer Engineering Department, University of Technology- Iraq, Baghdad 19006, Iraq

**Keywords:** omics, deep learning, genomics, transcriptomics, metabolomics

## Abstract

This research aims to review and evaluate the most relevant scientific studies about deep learning (DL) models in the omics field. It also aims to realize the potential of DL techniques in omics data analysis fully by demonstrating this potential and identifying the key challenges that must be addressed. Numerous elements are essential for comprehending numerous studies by surveying the existing literature. For example, the clinical applications and datasets from the literature are essential elements. The published literature highlights the difficulties encountered by other researchers. In addition to looking for other studies, such as guidelines, comparative studies, and review papers, a systematic approach is used to search all relevant publications on omics and DL using different keyword variants. From 2018 to 2022, the search procedure was conducted on four Internet search engines: IEEE Xplore, Web of Science, ScienceDirect, and PubMed. These indexes were chosen because they offer enough coverage and linkages to numerous papers in the biological field. A total of 65 articles were added to the final list. The inclusion and exclusion criteria were specified. Of the 65 publications, 42 are clinical applications of DL in omics data. Furthermore, 16 out of 65 articles comprised the review publications based on single- and multi-omics data from the proposed taxonomy. Finally, only a small number of articles (7/65) were included in papers focusing on comparative analysis and guidelines. The use of DL in studying omics data presented several obstacles related to DL itself, preprocessing procedures, datasets, model validation, and testbed applications. Numerous relevant investigations were performed to address these issues. Unlike other review papers, our study distinctly reflects different observations on omics with DL model areas. We believe that the result of this study can be a useful guideline for practitioners who look for a comprehensive view of the role of DL in omics data analysis.

## 1. Introduction

Omics refers to the branch of study in biological sciences that end with “-omics,” such as genomics, transcriptomics, proteomics, or metabolomics. The suffix “-ome” refers to the research subjects in domains such as the genome, proteome, transcriptome, or metabolome. Omics studies mainly aim to identify, describe, and quantify all biological molecules involved in the structure, function, and dynamics of a cell, tissue, or organism.

Multi-omics research flourishes in the areas of genomes, proteomics, transcriptomics, microbiome, metabolomics, pathomics, and radiomics [1]. Researchers have paid close attention to the connection between multi-omics data, medications, and illnesses. Moreover, multi-omics may accurately predict the diagnosis, prognosis, and course of treatment for illnesses. At various levels, a network may represent study entities, including genes, RNAs, proteins, microorganisms, metabolites, pathways, and pathological and medical imaging data. Additionally, some computer science and biology researchers have attempted to employ computational techniques to investigate potential connections between biological elements [2]. Instead of single-omics data, multi-omics data can improve the performance of these techniques because they obtain a vast landscape for understanding biological systems and mechanisms. However, heterogeneous data from multiple sources result in high complexity and different kinds of noise, which are detrimental to information extraction [3].

For instance, the median overall survival for tumor patients receiving the current medical standard of care is three years [4]. This outcome is due to the high level of heterogeneity in any form of tumor and the complicated biological molecular markers. Therefore, the community urgently needs the development of computational techniques for identifying novel treatment targets. Numerous attempts have been made to determine the molecular subtypes of gliomas and understand the heterogeneity across tumor types [4]. Numerous new molecular subtypes have been discovered based on several data sources, including histology, gene expression patterns, and driver genes [5,6,7,8]. However, applying the existing subtypes to clinical practice is challenging because the survival prognosis in the study of molecular subtypes is barely considered and has poor performance. The consideration of survival time is crucial in classifying tumor patients into useful subcategories with varying prognoses. Additionally, identifying community-wide high-risk tumor subtypes as appropriate treatment targets remains difficult. Consequently, an urgent need arises for computational methods to categorize patients accurately into subgroups linked with survival and identify prospective treatment targets.

Many computational methods have been developed with the rapid development of computer technology. Many research methods on intermolecular associations of humans related to different diseases, such as heart disease [9], Parkinson’s disease [10], hearing loss [11], and COVID-19 [12,13,14], were developed. Big data and artificial intelligence technologies are expected to drive the rapid development and modernization of traditional Chinese medicine [15]. Machine learning (particularly deep learning [DL]) technologies play an active role in computational biology; they also gain remarkable success in biological fields with the advancement of artificial intelligence and high-performance computing [16,17,18,19]. For instance, DL has been used in identifying genetic variations, DNA methylation, and image analysis, thereby leading to remarkable advancements in these areas [4]. DL is a promising subject when used with data from various omics. However, the following factors often result in limitations of current techniques. The majority of multi-omics approaches concentrate on two to three data kinds with a narrow scope for a specific sample or patient because of the growing number of accessible data types. Unsupervised learning has poor interpretability in anticipated outcomes or classifier labels [4].

On the basis of the above concepts, we introduced a comprehensive study covering all aspects of omics data that comprise the DL approaches. All research articles were scanned from two perspectives: technical and medical perceptions. The healthcare research field has enormous potential and problems because of the widespread availability of complex and unstructured omics data. Thus, the literature on omics data must be investigated thoroughly. The review paper’s contributions and uniqueness are as follows:

- The development studies are highlighted to improve the medical processes based on DL models.
- Notable achievements by other researchers in response to omics needs are summarized.
- The real benefits of using DL models in omics data analysis are emphasized.
- The current challenges in DL models when used with omics data are clarified.
- Using DL, we provide a taxonomy that organizes the corpus of already-published materials and specifies several omics research trajectories. We believe that the findings are useful to other researchers.
- The challenges associated with using omics data in DL-based clinical applications, such as the high dimensionality and sparsity of omics data, the lack of labeled data, and the need for robust evaluation methods, are identified.
- The importance of interpretability, generalizability, and robustness in DL models applied in the omics field is highlighted.
- The ongoing research on using DL algorithms for omics data analysis and the integration of multi-omics data to improve disease outcome prediction are analyzed.

This paper is organized into different sections. Section 2 introduces the details of the systematic review procedure. Section 3 provides the results of the adopted systematic review protocol. Section 4 focuses on the existing datasets used for omics data analysis. Section 5 highlights the challenges linked to omics studies when DL approaches are used. Section 6 concludes the contributions of the present study and maps the addressed challenges with achieved outcomes.

## 2. Systematic Review Protocol

### 2.1. Information Sources

We selected four of the most popular online search engine databases for a systematic search: Web of Science (WOS), ScienceDirect (SD), PubMed, and IEEE Xplore Digital Library. The selection was made by following an index that simplifies the formulation of complex search queries and keeps track of numerous scientific journals and conference papers in computer science, biology, medicine, biomedical engineering, and biomedical computing. We aimed to include as much material as feasible and as many articles on Omics data and DL as possible in the selection. We also aimed to provide a holistic view of researchers’ achievements in a wide but pertinent variety of disciplines.

### 2.2. Study Selection

**Figure 1:**
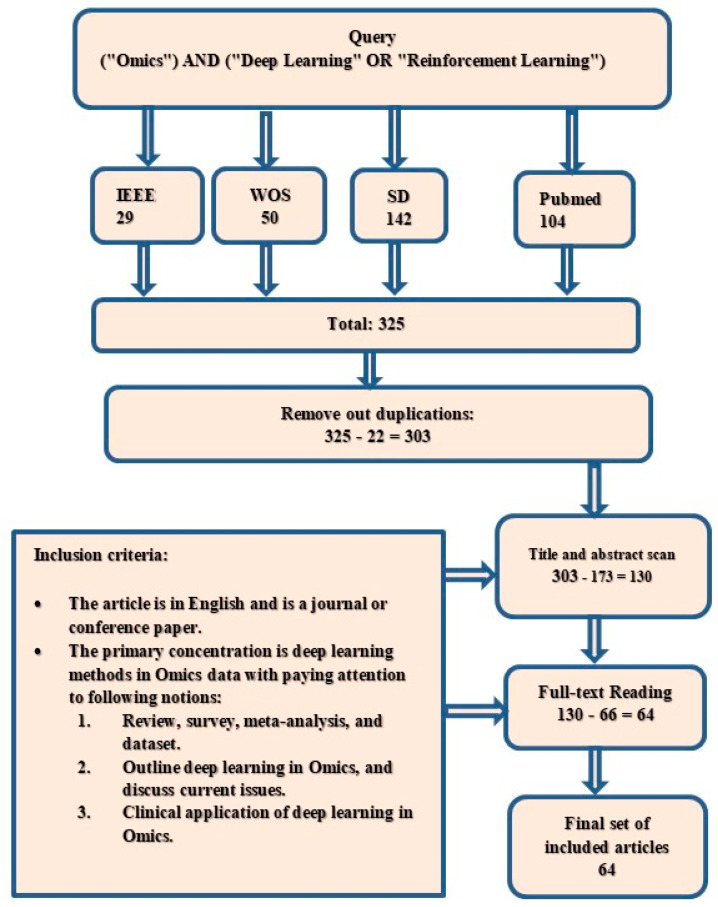
Study selection protocol.

The research selection method required a thorough two-step search of linked literature. Reading the titles and abstracts enabled the initial removal of duplicated and irrelevant items. Second, full-text reading was used to filter the articles scanned in the preceding stage. The same procedures were used for both levels. Figure 1 shows the study selection protocol used in the present work.

### 2.3. Searching Settings

The article search procedure was conducted on 18 April 2022. The search query was entered into the search boxes of the IEEE, WOS, PubMed, and SD databases. The searches were conducted in all of the aforementioned databases using terminology-related keywords (“Omics”). Afterward, these keywords were paired with the keywords “Deep Learning” or “Reinforcement Learning” using the “AND” operator, as shown in Figure 1. All authors agreed that the selected query and search engines were suitable for the search process. The most relevant articles were obtained during this process. Each search engine’s advanced search settings were used to limit the search to relevant journals and conference papers and exclude book chapters and other sorts of publications, such as white papers and workshops. We also looked at papers that were clearly based on the most recent and appropriate scientific research relevant to DL in omics data analyses.

### 2.4. Eligibility Criteria

**Figure 2:**
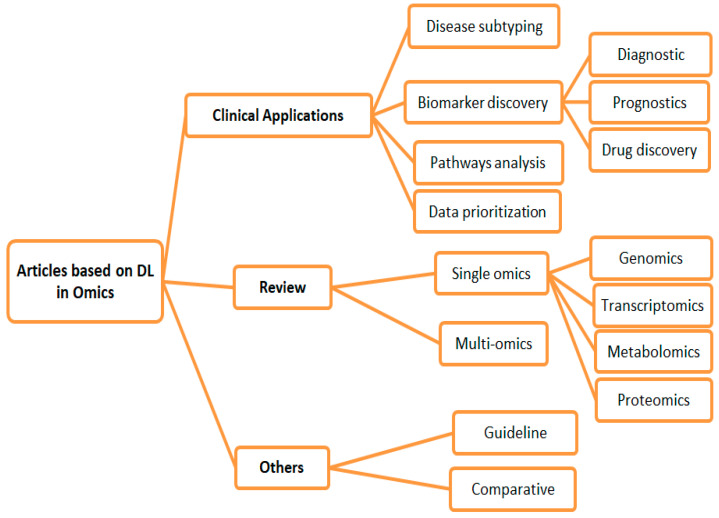
Proposed taxonomy.

All articles that match the criteria shown in Figure 1 were included. We set the primary goal as mapping the compass of research on omics into a wide-range and coarse-grained taxonomy of three groups. After a thorough preview of the available literature, the groups were chosen without limitation. We eliminated the articles that did not meet the qualifying requirements to remove duplicate articles. The following points comprised the exclusion criteria: (1) The article is not in English. (2) Omics and DL are briefly discussed. Figure 2 shows the proposed taxonomy.

## 3. Results

The details of the first run of the search query filtering 325 articles are as follows: 29 articles from the IEEE Xplore search engine, 142 articles from SD, 104 articles from PubMed, and 50 articles from WOS over a period of five years (2018–2022). The number of duplicate articles was 22. A total of 130 articles were left after 173 were excluded for being unrelated via title and abstract scanning. After the review of all the manuscripts, 66 articles were eliminated. The final group of articles eventually included 64 articles. These publications were carefully read to obtain a general research overview of this new field. However, several studies concentrated on the same topic. The articles were sorted into groups according to the study goals and used in the taxonomy procedure. Figure 2 shows a potential taxonomy for evaluating studies applying DL in omics. The acquired taxonomy may be broken down into three distinct article categories. First, only 35 of the 64 publications focused on the research on DL as a clinical application in the omics field. These publications include studies on disease subtyping, biomarker discovery, pathway analysis, and omics data prioritization. Second, 22 studies conducted a review and survey. They reviewed two aspects: single omics and multi-omics. Third, seven articles focused on other aspects of omics data, such as comparative analysis and guideline studies. We identified the general types of articles via summaries. Then, we changed the categorization into a literature taxonomy, as shown in Figure 2. We also illustrated numerous distinct subcategories of the primary classes in this diagram without any overlap. The following sections describe the recognized categories and condensed data related to them.

As illustrated in Figure 2, we rebuilt the categorization into a literary taxonomy indicating the broad types of articles through outlines. Multiple nonoverlapping subclasses of the primary classes are also depicted below. The sections that follow detail various perceptions and provide concise data for support.

### 3.1. Clinical Application Studies

Clinical application (development) papers (35/64) focused on enhancing the process of diagnosis, prognostication, therapy, and knowledge discovery. They comprised the majority of research works on omics data using DL. We categorized development articles according to the case study information offered in the literature, particularly in the parts of datasets and findings.

In general, precision medicine seeks to replace generic treatments for a large population with customized, targeted medications and treatment plans based on each patient’s molecular profile [20]. Alternatively, it seeks to develop preventative medicine plans using disease susceptibility assessment [21]. Omics data are crucial to this shift because they allow for the simultaneous analysis of illnesses at several levels (such as the DNA sequence, gene expression, and medical imaging). Moreover, the specific elements of the affected complex biological activities can be identified. Several ML-based technologies have been used in this new context to apply medicine [22].

#### 3.1.1. Disease Subtyping

Disease subtyping is relevant to studies aimed at finding groups of patients who exhibit different therapeutic/prognostic outcomes [23]. Moreover, disease subtyping concerns diseases comprising multiple subtypes implicated in prognosis [24]. Disease subtyping is vital for diagnosis and individualized patient therapy [25]. In particular, 5 out of 35 articles on the disease subtyping based on DL models were found, as shown in Table 1.

Computational multi-omics approaches are based on DL techniques; they typically aim to classify patients according to disease subtypes [26]. These methods are usually exploited to detect regularities and patterns revealing different disease molecular subtypes or disease classifications that share a common pattern of pathway perturbation [27]. The most common disease is cancer; it is divided into several categories according to various criteria, such as phenotype, molecular portraits, and histopathology [28]. Several works in this direction have been proposed using direct DL classification models or clustering methods. Cancer is the most common disease that requires solution subtyping. According to the authors in [26], next-generation sequencing (NGS) methods accelerate human genome mapping. Sophisticated NGS methods indicate that several genetic molecules are involved in developing breast cancer and its subtypes. Consequently, a DL model based on a stacked autoencoder (SAE) is needed to diagnose different forms of breast cancer accurately. In the present study, the authors used multi-omics data, such as miRNA, mRNA, and DNA methylation, which are pivotal in breast cancer subtype classification. They also play a crucial role in breast cancer formation. Moreover, the authors concluded that SAE could reduce the dimension of high-dimensional multi-omics data. In [29], the authors highlighted that most of the existing methods for breast cancer subtyping used only gene expression to identify cancer subtypes; another major issue is that most of the existing clustering methods completely ignore the results from prior knowledge. Thus, the authors designed a new DL fusion clustering framework to integrate multi-omics data (mRNA expression, miRNA expression, and DNA methylation) with The Cancer Genome Atlas (TCGA) BRAC dataset for breast cancer subtype identification.

Table 1 shows that subtyping can be done via a classification task, clustering process, or both. The most frequent DL model for such a process is the autoencoder (AE) model and its improved versions. However, the data type most frequently used for classification or clustering type is miRNA and mRNA. However, data subtyping is used mainly for cancer disease subtyping, particularly breast cancer.

**Table 1:** Disease subtyping articles.

Ref.	DL Task	DL Name	Omics Data	Disease Type	Statistical Test Method	Outcomes	Implementation Source
[26]	Classification and reduction of high-dimensional multi-omics data	SAE	miRNA, mRNA, and DNA methylation	Breast cancer	Bonferroni corrected the *p*-values of the *t*-test	Accuracy = 92%	http://www.nitttrkol.ac.in/indrajit/projects/integrated-analysis-breastcancer-subtypes/ (accessed on 23 December 2022)
[29]	Clustering and classification	SAE and AE	mRNA expression, miRNA expression, and DNA methylation	Breast cancer	*p*-value and *t*-test	Silhouette score = 0.664	-
[24]	Data classification	Variational AEs (VAEs) and feedforward neural networks	RNA-seq gene expression (denoted as RNA below), miRNA expression, and somatic copy number alteration (CNA) data	Breast cancer	*p*-value and *t*-test	Accuracy reaching 94%	https://github.com/DEIB-GECO/brca_subtyping (accessed on 23 December 2022)
[30]	Data clustering and classification	Principal component analysis (PCA), MFA, and disjointed deep AE	miRNA expression, mRNA expression, and reverse-phase protein array expression	Breast cancer and neuroblastoma (NB)	-	-	-
[31]	Data classification	Graph Convolutional Network (GCN)	Gene–gene interaction (GGI) networks, protein–protein interaction (PPI) networks, or gene co-expression networks	Cancer	-	84% each for accuracy, precision, recall, and F-score	https://github.com/NabaviLab/GCN-on-Molecular-Subtype (accessed on 23 December 2022)

#### 3.1.2. Biomarker Discovery

Biomarker discovery involves studies that detect omics characteristics that indicate a disease state [23]. Biomarkers, sometimes referred to as biological markers or biomarkers, are measurable and evaluable indicators of specific biological states in healthy and pathological processes and potential pharmacologic reactions to therapeutics [32]. As one of the most prevalent categories, molecular biomarkers are extensively researched across many disciplines. Examples include important genes, RNAs, proteins, and metabolite molecules from tissues, blood, and other bodily fluids [33].

The discovery of novel biomarkers for early illness diagnosis, therapy response, and categorization is one of the most popular uses of omics technology in biomedical research. TCGA [34] and other publicly available omics datasets focusing on cancer allow the identification of novel biomarkers via DL [22].

Molecular biomarkers are found by analyzing the information supplied by various omics [35]. For instance, a precise and quantitative risk assessment for cardiovascular disease is provided by a high-sensitivity C-reactive protein test [36]. The planning of preventative actions and patient decisions is mainly influenced by biomarkers, which may be categorized as either diagnostic, prognostic, or predictive [37]. Prognostic biomarkers offer information on the overall prognosis with or without the standard course of therapy. In comparison, diagnostic biomarkers are used to determine the presence of illness in a patient. Before the onset of their physiological symptoms, biomarkers may help identify high-risk patients. They also help determine how a condition progresses [38,39]. In the present study, we considered that prognostic, predictive, survival analysis, and disease recurrence have the same meaning.

Drug discovery/repurposing research aims to find new medications or potent ones that are already on the market and created for other ailments [23]. The traditional drug discovery approach is dominated by target-based high-throughput screening. For decades, it has been the focus of computer-aided drug discovery, including the recent DL applications [40]. Precision medicine depends critically on the accuracy of prediction models that forecast the response of medications to treatment based on the molecular profiles of patients. Prediction models provide options for choosing acceptable individuals for clinical trials; they also assist physicians in choosing the most successful therapy option [41]. The objective of biomarker discovery research was emphasized in our study based on many factors, including data type, illness type, analytic techniques, and the presence of medical validation. The final number of articles in the indicated category is 22 out of 35, as shown in Table 2.

According to the above table, most biomarker discovery studies were conducted for prognostic purposes. Moreover, many studies on diagnostics of different case studies exist. However, studies on solving the issues with patients’ treatments are few. A GNN model is often used for biomarker identification because GCNs use a different methodology by attempting to categorize network nodes based on node characteristics and network design. Similar to CNNs, GCNs gather information from adjacent nodes in a hierarchical manner; they may also function in a semi-supervised setting when labeled nodes are in short supply [63].

Given the data issues, a statistical test based on *p*-value and *t*-test has been widely used to measure the validity of used data and obtained results. Finally, we observed that few studies validated the results from a medical viewpoint. This observation may direct us to a question: how meaningful are these results for the medical sector?

#### 3.1.3. Pathway Analysis

Pathways analysis or knowledge discovery is essential in studies aimed at discovering the relation among -omics terms, such as genes or proteins, and measuring the influences of these terms on DL performance. Thus, this category looks for relationships of used omics data rather than outcomes of DL models for diagnostic or prognostic purposes. In particular, only 5 out of 35 studies were found to belong to the pathway analysis category, as shown in Table 3.

According to the above table, most data used for omics analysis are usually text or numeric. However, image data, such as CT images, can also be used for this purpose. In [66], the authors used CT images to map the features between medical images and gene expression profiles and quantify their correlations. For improvement, different studies took full advantage of DL methods and characterized lung cancer clinically at both genome and image levels. However, medical validation can be directly from doctors or based on a validation method that applies only to the medical field. Adding clinical information, such as gender, age, stage, and smoking history, can also be used to validate the results of DL from a medical point of view. However, only a few studies conducted medical tests to show how vital the obtained results were for the medical sector.

#### 3.1.4. Omics Data Prioritization

The enormous amount of omics data offers a previously unheard-of potential for computational algorithms to prioritize illness candidates, thereby illuminating the course of human diseases and considerably facilitating cancer prevention, detection, and therapy. Thus, we discuss this trend in this section even though it demands some work. The three methods of prioritization are as follows: prevention, diagnosis, and treatment prioritization or gen prioritization.

The authors of [70] introduced a computational technique called MRSLA to find disease-associated miRNAs. The disease miRNA prioritization task was designed as a recommender system suggesting the miRNAs that tend to be responsible for a given disease based on a low-rank approximation framework. This robust AI algorithm can successfully incorporate multimodal features into the prediction model and produce a high-performing result [70]. In the same way, [71] utilized DL approaches for prioritizing complex disease loci by investigating the landscape of ML applications in three parts: selected models, input features, and output model performance.

On the contrary, the data analysis from several omics has indicated potential genes for measurable features. However, these data are not integrated well, particularly in nonmodel species. Thus, choosing candidate genes for further experimental confirmation becomes difficult. The authors of [72] utilized the DL method (CNN) that integrates multi-omics information to prioritize the candidate genes of objective traits.

### 3.2. Review Studies

#### 3.2.1. Single Omics

In this subsection, our study has focused on review papers that considered only a single type of common omics data. Mainly four types of omics directions have been investigated as follows:

##### Genomics

The study of the structure, function, evolution, and mapping of genomes is known as genomics. Its goal is to characterize and quantify the genes that control the creation of proteins with the help of enzymes and messenger molecules. Genomics uses techniques to analyze the DNA sequences for studying the structure and function of genomes, gene regulation, and genetic alterations that can be associated with several diseases [22].

We found further in the literature that several works have investigated this topic. In [40], the authors presented a mechanism-driven neural network-based approach, called DeepCE, for predicting the differential gene expression profile perturbed by de novo chemicals. DeepCE uses a GNN and multi-head attention mechanism to model chemical substructure–gene and gene–gene associations. Additionally, the authors suggested a unique data augmentation method that uses faulty trials in the L1000 dataset to extract relevant information. Extensive work was introduced in [73]. First, that work defined the objectives of learning perturbation response in single-cell omics (genomics). It also surveyed the existing approaches, resources, and cited datasets in GitHub and discussed how a perturbation atlas could enable DL models to construct an informative perturbation latent space. In the same work, the authors examined future avenues toward robust and explainable modeling using DNNs, thereby enabling the integration of disparate information sources and an understanding of heterogeneous, complex, and unseen systems.

The authors of [74] investigated how DL has contributed to and been utilized in the mining of biological data. Well-liked open-source DL tools relevant to these data and accessible open-access data sources related to the three data kinds were studied by concentrating on the application of DL to analyze data patterns from several biological areas. Additionally, comparative analyses of these tools from the qualitative, quantitative, and benchmarking viewpoints were presented.

Many ML- and DL-based metastatic prediction techniques created to date are summarized in the present article [75]. Various molecular data types utilized for the creation of models and crucial signatures obtained using various techniques were also described. Additionally, the authors emphasized the difficulties in using DL and ML techniques and provided recommendations to enhance their ability to anticipate outcomes.

In the utilization of genomics in Alzheimer’s disease (AD), [76] focused on the latest developments for AD prediction using DL techniques with the principles of neuroimaging and genomics. The authors first described several experiments that employ DL algorithms to develop AD prediction using genomes or neuroimaging data. They particularly outlined pertinent integrated genomics and neuroimaging studies that use DL techniques to predict AD by combining both genomic and neuroimaging data.

Finally, the authors in [68] aimed to help fill the gaps in data preprocessing and genome-wide association study (GWAS) methodologies by reviewing novel techniques and methodologies. The data preprocessing performed prior to a GWAS presents challenges in Hardy–Weinberg estimation, genotyping, and accounting for factors such as sample structure.

##### Transcriptomics

The collection of all messenger RNA molecules in one cell, tissue, or organism is known as the transcriptome. In addition to the chemical identities, it contains the quantity or concentration of each RNA molecule. Transcriptomics measures the degree of expression for each RNA transcript generated in a cell. Transcriptomics raw data are often processed to produce expression matrices, which are frequently the input of DL techniques. These matrices contain an estimate of each gene’s or transcript’s level of expression across many samples and situations. Transcriptomics applications span a broad spectrum, and DL has been effectively used in some of these applications [22].

In the theoretical literature review covering the transcriptomics keyword, we found only two articles during the search period. First, the authors presented the most recent methods for determining the link between learned latent and observed variables and the external phenotypes in [77]. Thus, the scholars improved the comprehensibility of the DL methods. They exhibited the usefulness of the proposed techniques in an application with single-cell gene expression data (transcriptomics). The relationships between observed gene expressions and experimental variables or phenotypes can be understood well because of the work presented here. Deep generative models may also provide synthetic observations by offering a generative model for the latent and observed variables, thereby enabling us to evaluate the uncertainty in the learned representations.

Second, the authors analyzed the most recent data modeling techniques used with transcriptomics data I TGx in [78]. The researchers demonstrated how the TGx data might be used for the benchmark dose study. Then, the approaches for reading across and modeling adverse outcome pathways were also reviewed here. Additionally, the authors addressed how network-based strategies may effectively define the mechanism of action or particular exposure biomarkers. Moreover, they explained the primary AI techniques used for developing predictive classification and regression models using TGx data while addressing the existing challenges.

##### Metabolomics

The metabolome represents the collection of all metabolites in a biological cell, tissue, organ, or organism. These metabolites are the end products of cellular processes. Metabolomics is the science that studies all chemical processes involving metabolites. In particular, metabolomics is the study of chemical fingerprints that specific cellular processes establish during their activity. It is the study of all small-molecule metabolite profiles.

We found several state-of-the-art works in this type of omics. A historical perspective work [79] introduced the readers to the fundamental computational concepts of ANNs. It also provided a brief historical context of their use in metabolomics, discussed the advantages and disadvantages of this method when applied to omics data, and finally looked toward future applications and challenges.

In [80], the authors explored breakthrough approaches for natural product discovery from plant microbiomes, emphasizing the promise of DL as a tool for endophyte bioprospecting, endophyte biochemical novelty prediction, and endophyte regulatory control. It concludes with a proposed pipeline for harnessing a global database to uncover and unsilence desirable natural products.

Finally, [81] presented the applications of DL that have recently emerged in metabolomics research. Then, the scholars presented the utilization of DL in the data preprocessing step. They also reviewed the use of the CNN model in developing DL for metabolomics data.

##### Proteomics

The term *proteome* refers to the sum of all the proteins in a cell, tissue, or organism. Proteomics is the science that studies these proteins in terms of their biochemical properties and functional roles. How the quantities, modifications, and structures of these proteins change during growth in response to internal and external stimuli was also investigated.

In this area, we found only one article that covered proteomics data. This scenario is related to the natural behavior of researchers because protein is usually used with other omics data to detect many tumors and cancers; moreover, protein is not used in single omics except in a few cases, including [82]. In the same work, the authors demonstrated the effectiveness of ensemble learning for reliable protein abundance prediction using single-cell multimodal omics data. Their study paved the way for knowledge discovery by mining single-cell multi-omics data on a large scale. Their work was accomplished by contrasting numerous tree-based ensemble learning techniques with neural network models. According to their work, ensemble learning frequently outperformed neural networks. In particular, random forest (RF) exhibited the greatest overall performance. Moreover, they interpreted the biological mechanisms driving the prediction using the feature significance ratings from RF.

#### 3.2.2. Multi-Omics

As we mentioned above, multi-omics currently has promise for filling the gaps in the understanding of human health and disease. Many researchers are working on ways of generating and analyzing disease-related data. They also describe the relations between omics data to predict the diagnosis, prognosis, and treatment of diseases. In this section, we reviewed the survey articles working on all previously mentioned aspects, as shown in Table 4.

As shown in Table 4, different types of literature papers have been mentioned in the second column as review, survey, systematic review, comprehensive review, methodological review, and system biology review. Sequentially, The Survey paper gives information about the amount of research done so far in a particular domain or on topic. In contrast, the Review paper analyzes published works and provides insights with technical evidence. A review article or review paper is based on other published articles. It does not report original research. Where and how one searches for evidence is an important difference. While literature reviews require only one database or source, systematic reviews require more comprehensive efforts to locate evidence. Multiple databases are searched, each with a specifically tailored search strategy (PRISMA). A systematic review that is less rigorous is often called a “Comprehensive review”. On the same side, a methodological review is a type of systematic review that focuses on summarizing the state-of-the-art methodological practices of research on a substantive or essential topic. Finally, a system biology review is a new type of paper that aims to provide specialists with a unique and educational platform to keep up-to-date with the expanding volume of information published in the field of Systems Biology, which belongs to a systems biology journal [83].

**Table 4:** Review and survey articles in the multi-omics area.

Ref.	Type of Literature Review	Omics Data				Models Reviewed	Disease Type	AI Model	
		Genomics	Transcriptomics	Metabolomics	Proteomics			DL	ML
[84]	Systematic review	√	√			CNN	Different cancer types	√	
[85]	Survey	√	√	√	√	RF, AE SVM, CNN, RNN, and MLP	General cancer	√	√
[86]	Review	√	√	√	√	CNN and other DL neural networks	Head and neck tumor	√	
[87]	Review	√	√			CNN and other DL neural networks	Cancer diagnosis	√	
[1]	Comprehensive review	√	√	√	√	-	Different diseases	√	
[88]	Review	√	√		√	CNN, DNN, ANN, RNN, and AE	Alzheimer’s and Parkinson’s diseases	√	
[89]	Systematic review	√	√	√	√	-	-	√	
[90]	Method review	√	√	√	√	-	COVID-19	√	√
[83]	A systems biology review	√		√		-	Hallmarks of cancer	√	
[91]	Review	√	√		√	DNN and RNN	-	√	√

We found four types of reviewing and survey articles in the literature about multi-omics areas with DL approaches. Some of the omics data take up all four omics data. Some of them only take two of the omics data. As shown in Table 4, the DL models included in the articles varied from not mentioning any model and confining to mentioning DL in general to mentioning six DL methods and their relationship with multi-omics. The target disease type must be mentioned in the review articles. Some of the works dealt with cancers and tumors in general. Some of them identified a specific type of disease and the works that dealt with this disease. From the above, we can summarize that review and survey works are vital for researchers to understand the relationship of multi-omics with DL from two perspectives: medical and technical points of view.

### 3.3. Others

Some works were not classified under development and could not be considered a review of previous research. Therefore, we decided to classify them as follows.

#### 3.3.1. Guidelines

Guideline papers aim to simplify the processes of utilizing DL in multi-omics according to a routine or sound practice. The three types of guideline papers are proposal, perspective, and narrative papers. They serve the same purpose in terms of presenting ideas. In addition, the articles’ main objectives differ. Table 5 shows all types of guideline papers.

#### 3.3.2. Comparative Studies

Comparative studies answer the question of which DL model is accurate in omics data. This objective was attained by comparing two or more DL models with single- or multi-omics data based on the metrics shown in Table 6.

## 4. Current Omics Datasets

The cornerstone of conducting an omics analysis is the availability of a dataset. In general, a dataset can support omics or multi-omics information. Moreover, this dataset can be associated with a single disease or multiple diseases. However, most omics datasets are not injected directly into a computational model unless a little preprocessing is performed for data enhancement or integration. In the present review, we tried to cover the existing datasets with details links to data type, number of samples, disease type, and implementation links, as shown in Table 7. In particular, all existing datasets are different in terms of the number of samples and the frequency of usage. For instance, we found that the cases in TCGA are the most frequently used data by omics studies. Moreover, the number of samples in this dataset is the largest. Moreover, TCGA is preferred by researchers because of the availability of tools for the required analysis and different types of omics data.

## 5. Challenges

**Figure 3:**
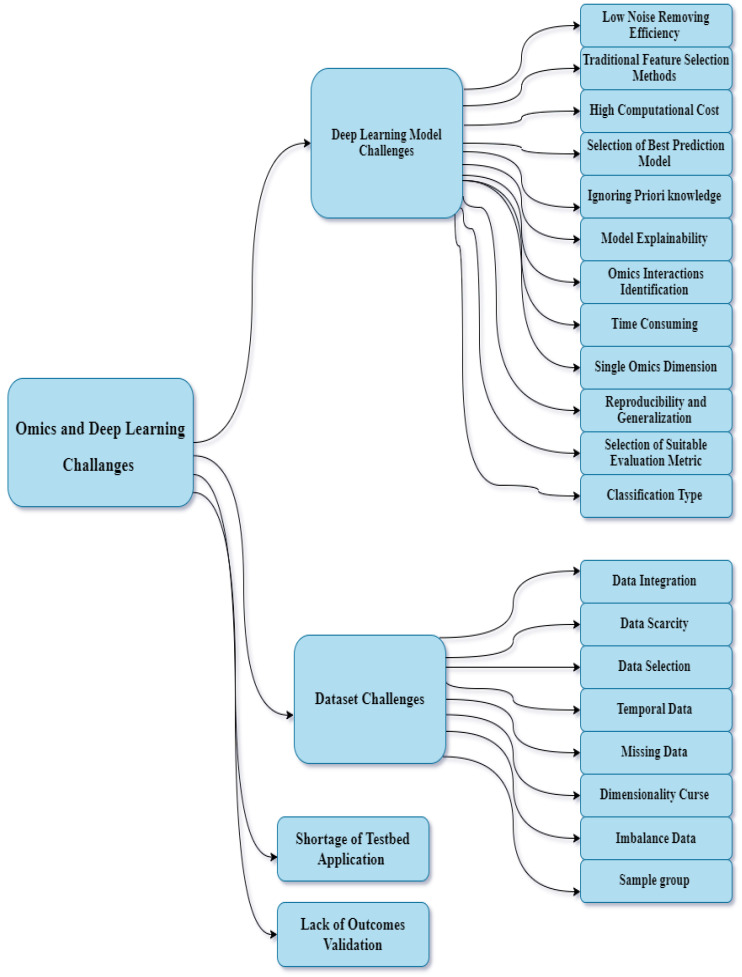
Omics and DL challenges.

In general, DL models link various challenges that affect the outcomes of classification or regression processes. In the DL models employed for omics analysis, we discovered many DL-related issues. The preprocessing procedure, datasets, model validation, and testbed applications are discussed in the following subsections (see Figure 3).

### 5.1. DL Model Challenges

#### 5.1.1. Low Noise Reduction Efficiency

The most popular experimental method in proteomics is separating proteins into short amino acid chains (peptides) and then using an MS to evaluate these peptides. The MS output signals are matched with the peptide profiles kept in open or private databases to identify the peptides. These databases are still unreliable. They also lack information. Additionally, DL is used to infer protein secondary structures from their amino acid sequences [108]. The creation of data for proteomics and metabolomics depends on NMR technologies. Given its technological limitations, it often returns noise signals that must be filtered to increase accuracy. Using DL approaches to metabolomics data is particularly difficult because identifying unique components contributing to individual samples in these kinds of research is crucial [22,109].

#### 5.1.2. Traditional Feature Selection Methods

The authors in [51] suggested using techniques based on DMPs and DEG to train models. These authors did not employ traditional feature selection or dimension reduction methods, such as LASSO, Relief-F, or PCA, because they cannot capture the bifurcated processes logically. This finding implies that using these techniques may result in few characteristics or dimensions; however, the occurrence of a few features with biological significance is not ensured. Moreover, these methods are unsuitable for application to the multi-omics dataset. The interaction between two various omics datasets cannot be considered by these approaches. One biological factor that may regulate and govern the level of expression of genes adjacent to the CpG site is DNA methylation, which is particularly well-known. Conventional feature selection or dimension reduction methods cannot handle this trait [51].

#### 5.1.3. High Computational Cost

In the early days, GNNs learn a node’s features by iteratively propagating information from the neighboring nodes until convergence [110]. High computing costs and the learning filters’ lack of the localization attribute are two notable drawbacks of these models. Chebyshev polynomials were proposed by Defferrard et al. in 2016 as localized learning filters in a spectral-based GCN (ChebNet) for converting computing costs into linear complexity [31,111].

#### 5.1.4. Selection of Best Prediction Model

Different omics applications have generated several prediction algorithms. These applications are founded on various computational techniques and diverse omics data sources. However, several studies [112,113] have demonstrated that various factors affect how well prediction algorithms function. We must first discover the important criteria affecting the performance of these algorithms to choose an appropriate prediction algorithm for a certain application. Then, we can utilize the identified factors to suggest a prediction algorithm for the application [52].

However, extensive training, testing, and cross-validation are required to determine the most appropriate weights for each parameter and its influence on algorithm performance. Nonetheless, the efficacy of unstable techniques varied based on the combined omics profiles, drugs, and performance indicators. The evaluations of these methods depend on (1) performance measurements and (2) supported data (e.g., omics characterization integrated into the model and drug structure/pathways). Thus, a difficult issue is to integrate different existing prediction models and determine which model is most effective in each scenario [47]. However, the application of CNNs to omics data poses several obstacles, such as the processing of complicated network architectures and its integration with transcriptome data [49].

However, the kind of model architecture limits how the information may be integrated. For instance, in the bioinformatics literature, a feedforward NN called a visible neural network can only represent directed acyclic graphs, which do not match with some knowledge networks (KEGG and STRING). Additionally, only the first hidden layer is connected to the input genes; the subsequent hidden layers are unrelated. The connections between failing levels are also removed. Consequently, a piece of information must be shortened to be absorbed into the neural network [56].

#### 5.1.5. Ignoring Prior Knowledge

Most of the existing clustering methods ignore established results from prior knowledge. Prior biological knowledge (e.g., Pam50 breast cancer subtypes) can guide representation learning. According to the gene expression profile analysis [114], breast cancer was first classified into five intrinsic subtypes according to the Pam50 breast cancer subtyping: Luminal A, Luminal B, basal-like, normal-like, and HER2 [29].

#### 5.1.6. DL Model Explainability

The lack of interpretability of such approaches is one of the most difficult issues impeding the growth of ML in healthcare. Most ML techniques, including DL techniques, are regarded as black boxes because of their complexity. The choices of these models cannot be explained. One of the most crucial current concerns is making ML algorithms interpretable. In the medical field, final users (e.g., researchers, clinicians, and patients) must understand why a phenotype has been predicted to ensure that it is based on reliable medical features rather than on irrelevant artifacts. This understanding has a considerable impact on their decisions and confidence in the model, regardless of the model’s efficacy. Finally, the model examination may aid biological discovery by exposing intriguing signs. The application of sophisticated machine learning models, including DL, on omics data, enables the emergence of precision medicine. However, their use in clinics is limited and not explainable [56].

Although DL approaches allow for the uncovering of hidden patterns in opaque and complex data, the models themselves are complex and opaque; thus, researchers cannot easily infer how learned latent representations relate to the observed variables [69]. Enhancing the interpretability of machine learning from a “black box” from genotype to phenotype is also challenging, particularly for uncovering causal mechanisms from risk variants to diseases [115].

#### 5.1.7. Omics Interaction Identification

Identifying the interactions and crosstalk between heterogeneous RNA classes is essential for uncovering the functional role of individual RNA transcripts, particularly for unannotated and sparsely discovered RNA sequences with no known interactions. As high-performing and adaptable techniques that can either predict RNA–RNA interactions from sequence or infer missing interactions from patterns that may exist in the network topology, sequence-based DL and network embedding methods have been gaining popularity. However, the majority of the approaches used today have several drawbacks, such as the inability to make inductive predictions, determine the directionality of interactions, or combine different sequences, interactions, expressions, and genomic annotation datasets [116].

#### 5.1.8. Time-Consuming

Matrix factorization (MF) methods are general models that use shared features across diseases and genes to predict genetic links between diseases [117]. However, MF-based algorithms often take too long to converge and can only handle a restricted variety of data during actual performance [55].

#### 5.1.9. Single-Omics Dimension

Most of the existing methods use only gene expression to identify cancer subtypes [29]. The individual type of omics data only represents a single view that suffers from data noise and bias [60,61]. Huge and various types of genetic data have been produced with the advancement of high-throughput sequencing techniques. The integration of multi-omics data contributes to cancer subtype identification.

#### 5.1.10. Reproducibility and Generalization

DL success depends on the random numbers produced at the beginning of training; moreover, practitioners may adjust hyperparameters, including learning rates, batch sizes, weight decay, momentum, and dropout probabilities [118]. Setting the same random seeds across many stages ensures the same experimental outcome. Although the hyperparameters and random seeds were often not provided in the present study, keeping the same code bases was crucial [119]. Reproducing the study and achieving the same results are challenging because of the uncertainty of the configuration and the randomness involved in the training [120].

#### 5.1.11. Selection of Suitable Evaluation Metric

Classification accuracy is often used for evaluating algorithm efficacy. However, it may be deceptive when used for data imbalance because an unbalanced training set may result in a classifier that favors frequent classes. In binary classification, the classifier may assign each test point to the dominant class and provide an optimistic accuracy estimate. BACC [121] was created to handle unbalanced training data (true negative rate) by averaging sensitivity (true positive rate) and specificity. In particular, BACC balances the contributions of different classes by giving each class a weight. Thus, the classifier produced can learn the same amount from each class. With binary predictions, BACC is also equal to the area under the ROC curve. This metric is the same as the regular accuracy if the dataset is balanced. Thus, the disease and control samples were not the same size in this application [115].

#### 5.1.12. Classification Type

The GCN is blind to different subpopulations within a cancer tissue because of the averaging of characteristics across patients for a specific cancer type. A possible drawback of the present situation is the vague idea of cancer genes. A multiclass, multilabel scenario becomes increasingly appropriate and enables the prediction of the cancer genes that are particularly unique to a single cancer type, thereby opening the door for precision oncology. In comparison, certain genes substantially affect numerous cancers [54].

### 5.2. Dataset Challenges

#### 5.2.1. Data Integration

Identifying disease-related lncRNAs is crucial for the diagnosis, prevention, and treatment of diseases. Many computational approaches have been developed; however, effectively integrating multi-omics data and accurately predicting potential lncRNA-disease associations remain challenging, particularly regarding new lncRNAs and new diseases [64]. In omics, several datasets comprise diverse data in terms of variable count, scale, distribution, and modality. The analytical goal in healthcare is challenging because of the heterogeneity of the data. Genetic instability is the primary contributor to heterogeneity [122], thereby complicating the determination of particular prognostic genes using only gene expression patterns [43]. Many machine learning techniques, including clustering, graph and network analysis, kernel learning, and DL, may be used in addressing the issue of heterogeneity [26,30].

The process of extracting heterogeneity from GRNs is another challenge. Previous works used GNN algorithms to combine homogeneous GRN with single-omics data. However, this approach is primarily ineffective in extracting the complex information present in heterogeneous GRN, particularly its rich variety of interconnection [44].

#### 5.2.2. Data Scarcity

In the field of brain-related diseases, the omics data obtained using advanced sequencing technology typically have few patient samples (tens to hundreds of samples) [123]. Reliably training complicated model architectures with limited labeled data remains a challenge. The scarcity of labeled multi-omics data may result in the overfitting/underfitting of complicated models, e.g., DL methods [44].

#### 5.2.3. Data Selection

Millions of genes have been sequenced in the data-driven genomics era; however, their causal relationships with disease phenotypes remain limited because of the difficulty of elucidating underlying causal genes using laboratory-based strategies [67].

#### 5.2.4. Temporal Data

The illnesses in healthcare evolve and change over time in a non-deterministic way. Machine learning algorithms have trouble dealing with the time component because they assume that inputs are static [124,125].

#### 5.2.5. Missing Data

Missing data refers to observations that are absent for various reasons, such as a protein’s decreased sensitivity or inability to process NGS data. When the majority of the samples in a dataset have missing data, the missingness becomes problematic. This issue is solved by list-wise deletion, which removes the whole sample of missing variables from the dataset.

However, the approach can result in loss of information if the percentage of missing data is large. Another option is imputation, which assigns the variable with missing data some random values, often the mean or median. The k-nearest neighbors method is mainly used to impute missing data [91,124].

Furthermore, a study defining the specific ratio for excluded features with missing data remains lacking. For instance, the authors in [61] eliminated the features that were absent from each cancer dataset in more than 20% of the patients, in addition to excluding patient samples if these data missed more than 20% of the remaining multi-omics characteristics. Then, the authors eliminated the cancer datasets that included less than 50 unfiltered samples.

#### 5.2.6. Dimensionality Curse

Most healthcare datasets include few samples but many characteristics or variables. Overfitting becomes an issue for many types of machine learning algorithms as the number of variables increases. Imaging, clinical, and omics data are just a few examples of the many kinds of information that may be found in healthcare databases. Various data reduction methods must be performed to make high-dimensional datasets suitable for learning. Feature extraction and feature selection methods are commonly used [126].

PCA and non-negative matrix factorization are two examples of the types of analyses utilized in feature extraction. Feature extraction plays a central role in unsupervised learning, in which answer labels are unknown. Machine learning with feature extraction is commonly utilized in multi-omics data to identify disease groupings. Selected features are used in feature selection. One can pick features using a filter, a wrapper, or an embedded method. Unlike other feature selection techniques, the filter chooses a subset of characteristics independently of any model. Pearson correlation, ANOVA, and information gain are just a few filtering techniques that may be applied. The wrapper technique uses a prediction model to select the most useful traits iteratively while retaining the least useful ones. Boruta, Jackstraw, and Recursive Feature Elimination are examples of the many wrapper techniques that may be applied. Wrapper approaches perform computation on massive datasets inexpensively. The embedded technique falls between the filter method and the wrapper method in terms of computational complexity. LASSO is an embedded technique. Supervised learning is mainly used for feature selection in classification and regression. Not all features are meaningful in high-dimensional datasets; thus, feature selection methodology is utilized prior to dataset integration [43,48].

#### 5.2.7. Imbalance Data

In healthcare, an imbalance of data occurs when the samples with positive values in the target class in the dataset are less than those with negative values.

Several techniques can address the imbalanced data in machine learning, including data sampling, algorithm modification, and ensemble learning. The dataset is balanced before the machine learning for classification in sampling is utilized. Undersampling and oversampling are typically used together to address the problem of an unbalanced dataset. When algorithms are changed, the same dataset with imbalance is used to make the changes [59]. Giving samples with minority types of high weights modifies the algorithm. In ensemble learning, the aggregate of all classifier judgments is obtained by applying each model subset of the majority of examples and considering all minority data [91,124,127].

#### 5.2.8. Sample Group

Most of the integrated molecular studies do not use the multi-omics dataset from the same sample group. Unlike the TCGA database, no database has produced considerable omics data from one sample [51].

### 5.3. Lack of Outcome Validation

Various DL-based single- or multi-omics publications have emerged in the past five years. The evaluation process has usually been conducted based on well-known measurements in the data science field, such as AUPRC, AUC, precision, recall, F-score, and ROC. These metrics can reveal how meaningful the obtained results are from the theoretical perspective. Most of the reviewed articles in this study have this kind of evaluation but lack supportive validation based on the medical perspective. However, the question of how remarkable and satisfying these results are for the medical sector ensues. Therefore, defining the measurements and procedure is essential for achieving the purpose and providing reliable results.

### 5.4. Shortage of Testbed Application

Many proposed works have reviewed omics data analysis using DL. However, no study has been deployed into medical infrastructure and translated into clinical practice.

One of the key issues hindering the clinical deployment of DL methods is transparency. A transparent and explainable ML algorithm seems essential to building trust for clinical decision-making [39,128]. Moreover, this deployment is missing because of the gap in consistent result validation between theory and clinical perspectives.

## 6. Conclusions

In the past decade, researchers have drawn considerable attention to the involvement of DL in omics data analysis, thereby making the DL and omics data one of the major research topics. In this context, no clear boundaries have been observed in the development of this field. Thus, further study is necessary to provide a holistic view and track this research line. Our study attempts to provide an extensive view and deep understanding by reviewing and classifying the highly pertinent literature. Consequently, this study maps the final set of relevant articles in three main categories: clinical applications, reviews, and other studies conducted using DL and omics data. In addition to offering an extensive investigation of the body of literature, the three main classes of articles are divided further into subcategories: such as disease subtyping, biomarker discovery, pathway analysis, data prioritization, review articles conducted to single- or multi-omics data, comparative analysis, and guideline studies. Other information is provided, including the difficulties and limitations of DL models and the information on the data and validation procedure. In the present review study, we found that the clinical applications that use DL in addressing various medical problems have received considerable attention from researchers. Additionally, the existing literature shows minimal concern for DL characteristics, feature data, and definition for a straightforward medical validation approach. Furthermore, testbed DL applications used in real-world settings or accepted by healthcare organizations are uncommon. Thus, the omics community should make considerable efforts to close the gap between theoretical and application viewpoints by creating practical applications. Finally, this systematic review assists researchers in tracking the crucial concerns about DL in omics by expanding and outlining new research avenues. In the future, the authors intend to extend this review by focusing on data integration methods and issues relevant to the existing methods.

## Figures and Tables

**Table 2:** Biomarker discovery articles.

Ref.	Aim	DL Name	Omics Data	Data Preprocessing Tools	Disease Type	Statistical Test Method	Outcomes	Implementation Source	Medical Validation
[42]	Prognostic	GCN	mRNA, copy number variation (CNV), and DN	-	Bladder urothelial carcinoma(BLCA), breast invasive carcinoma (BRCA), head and neck squamous cell carcinoma (HNSC), lower-grade gliomas (LGG), liver hepatocellular carcinoma (LIHC), lung adenocarcinoma (LUAD), lung squamous cell carcinoma(LUSC), ovarian serous cystadenocarcinoma(OV), sarcoma(SARC), skin cutaneous melanoma( SKCM), and stomach adenocarcinoma (STAD)	*p*-value and *t*-test	C-index = 0.652	-	No
[43]	Prognostic	Generative adversarial networks	Gene expression (mRNA), CNV, single nucleotide polymorphism, and DNA methylation	https://github.com/compgenome365/TCGA-Assembler-2 (accessed on 23 December 2022)	BRCA, acute myeloid leukemia(LAML), LIHC, LUAD, pancreatic adenocarcinoma(PAAD), STAD, and LGG of the brain	-	Compared with the Area Under The Curve (AUC) for the best-performing existing methods for seven cancer Types, the AUC for this method was improved by 4%.	-	No
[44]	Diagnostic	Graph attention network	mRNA, TF, and miRNA expression data	-	Breast cancer	-	ACC = 79%, macro-F1 = 78%, and micro-F1 = 81%	-	No
[45]	Prognostic	DeePROG	Gene expression profile, underlying DNA sequence, and 3D protein structures	-	Chronic lymphocytic leukemia(CLL), interstitial lung disease(ILD), and prostate	Welch’s *t*-test	F-score = 94.31, precision = 94.35, and recall = 94.35	https://github.com/duttaprat/DeePROG (accessed on 23 December 2022)	No
[46]	Prognostic	CapsNetMMD	mRNA expression, z scores for mRNA expression, DNA methylation, and two forms of DNA CNAs	-	Breast cancer	*p*-value and *t*-test	Specificity = 95, sensitivity = 75.8, precision = 85, recall = 89, AUC = 94.6	https://github.com/ustcpc/CapsNetMMD (accessed on 23 December 2022)	No
[47]	Drug discovery	Reinforcement Learning	DNA CNV, DNA methylation, point mutations, transcript expression, RNA sequencing, and protein abundance	-	Cancer therapy		F-score = 79, precision = 79, recall = 80, AUC = 98	https://github.com/salma2018/Q-Rank (accessed on 23 December 2022)	No
[48]	Diagnostic	VGG	Image (gene)	Random projection [30] and PCA [31]	Liver cirrhosis (CIR), colorectal cancer (COL), obesity (OBE), inflammatory bowel disease (IBD) and type 2 diabetes(T2D) [26]	*p*-value and *t*-test	-	-	No
[49]	Diagnostic	Customized convolutional neural network (CNN)	Gene expression profiles and binary PPI network	-	Lung Cancer	-	Specificity = 0.74, Precision = 0.78, Recall = 88, accuracy = 81	https://sites.google.com/site/nacherlab/analysis (accessed on 23 December 2022)	No
[50]	Prognostic	Graph neural networks (GNNs)	scRNA	Annoy’s method	COVID-19	-	Accuracy = 95.12%	https://github.com/nealgravindra/self-supervsed_edge_feats (accessed on 23 December 2022)	No
[40]	Drug discovery	GCN and multi-head attention mechanism	Gene expression profiles, PPIs, and information about drugs and their targets	Data augmentation	COVID-19	*p*-value, *t*-test, and Pearson correlation	-	https://github.com/pth1993/DeepCE (accessed on 23 December 2022) https://zenodo.org/record/3978774#.Yqbp7qhBxPY (accessed on 23 December 2022)	No
[51]	Diagnostic	Deep neural network (DNN)	Gene expression and DNA methylation profiles	Approaches based on the differentially expressed gene (DEG) and differentially methylated position (DMP)	Alzheimer’s disease	*p*-value and *t*-test	Accuracy = 83%	https://github.com/ChihyunPark/DNN_for_Adprediction (accessed on 23 December 2022)	No
[52]	Prognostic	ForgeNet model	Gene expression and miRNA and metabolomics dataset	-	Breast cancer	*p*-value and *t*-test	AUC = 74%	https://github.com/yunchuankong/forgeNet (accessed on 23 December 2022)	No
[53]	Diagnostic	Stacked Sparse Compressed Auto-Encoder	mRNA expression	-	Ovarian cancer and breast cancer	-	AUC = 98%	-	No
[54]	Diagnostic	GCNs	mutations, DNA methylation and gene expression data	HotNet2 [5] and ComBat [17]	Cancer	-	AUPRC = 83% and AUC = 88%	https://github.com/marcoancona/DeepExplain (accessed on 23 December 2022)	No
[55]	Diagnostic	Multimodal DBN	PPI and GGI	Goh et al. (2007); from the website of OMIM	Different types of diseases	-	AUC = 96%	https://github.com/luoping1004/dgMDL (accessed on 23 December 2022)	No
[56]	Diagnostic	Graph Neural Network (GNN)	Gene expression and RNA-Seq	-	Cancer	-	Accuracy = 95%,	-	Yes
[57]	Prognostic	AE	mRNA, miRNA, DNA methylation and CNVs	Lumi package in R and Gistic2.0	LUAD	*p*-value and *t*-test	C-index = 0.65, Log-rank *p*-value = 4.08 × 10^−9^	-	Yes
[58]	Prognostic	Graph neural networks	scATAC-seq data and scRNA-seq data	-	Cancer	-	Accuracy = 87%, balanced accuracy(BACC) = 95%	http://deeptfni.sysomics.com/, https://github.com/sunyolo/DeepTFni (accessed on 23 December 2022)	No
[59]	Prognostic	Deep belief networks	Gene, junction, isoform, miRNA, and methylation	mRMR (Peng et al., 2005) for the reduction of the dimensionality of input modalities; the uninformative features are removed	Kidney renal clear cell carcinoma (KIRC), HNSC diseases, and NB pediatric cancer	*p*-value and *t*-test	-	-	No
[60]	Prognostic	Transfer learning-based Cox proportional hazards network	R package “limma” and R package “imputeMissings”	RNA-seq, miRNA-seq, DNA methylation, and CNV data	Bladder cancer	*p*-value and *t*-test	C-index = 0.665	https://github.com/Hua0113/TCAP (accessed on 23 December 2022)	No
[61]	Prognostic	Denoising based on AE (DAE)	R package “limma” [22]	mRNA, miRNA, DNA methylation, and CNV	15 cancers from TCGA	*p*-value and *t*-test	C-index = 0.627	https://github.com/Hua0113/DCAP (accessed on 23 December 2022)	No
[62]	Prognostic	Multimodal DNN	mRMR [11]	Age at diagnosis, size, histological type, inferred menopausal status, positive lymph nodes, stage, mutation status, 400 features for gene expression, and 200 features	Breast cancer	-	Precision = 83%, Recall = 83%, accuracy = 83%	-	-

**Table 3:** Pathway analysis articles.

Ref.	DL Name	Omics Data	Data Preprocessing Tools	Statistical Test Method	Outcomes	Implementation Source	Medical Validation
[64]	Deep multi-network embedding (DeepMNE) model	Long noncoding RNA (lncRNA)	-	-	F-score = 87%, AUC = 94%	https://github.com/Mayingjun20179/DeepMNE (accessed on 23 December 2022)	No
[65]	Squeeze-and-excitation residual network and bidirectional gated recurrent unit	DNA sequences	-	*p*-value and *t*-test	AUC = 75%, accuracy = 67%	https://pubmed.ncbi.nlm.nih.gov/31161194/ (accessed on 23 December 2022)	No
[66]	U-Net and AE networks	CT scans and gene expression	-	-	Mean average error = 4.112 × 10^−6^, Mean square error = 4.318 × 10^−6^	-	Yes
[67]	Customized CNN	Gene expression and PPI	t-Distributed stochastic neighbor embedding algorithm and spatial vector representation, named ProtVec [68]	*p*-value and *t*-test	Precision = 74%, recall = 56%, accuracy = 70%	http://www.smartprotein.cloud/public/home (accessed on 23 December 2022)	Yes
[69]	Deep Boltzmann machines and VAEs	RNA-Seq data	Bernoulli distribution and DESeq	Cramer’s statistic and G-test of the goodness of fit	-	https://github.com/ssehztirom/Exploring-generative-deep-learning-for-omics-data-by-using-log-linear-models (accessed on 23 December 2022)	No

**Table 5:** Guideline articles.

Ref.	Year	Type	Ideas	Target
[92]	2019	Perspective	This perspective highlights key advances and challenges in precision oncology	Precision oncology
[93]	2019	Procedure	It includes the processes of AI-based integration of imaging, omics, and clinical data	General clinical applications
[94]	2019	Procedure	It describes the historical development and recent methodological advancements for studying disease classification	Classification of diseases (nosology)
[95]	2021	Proposal	This proposal shows how machine learning can identify sets of lipids as predictive biomarkers of nonalcoholic fatty liver disease progression	Diagnosis
[96]	2022	Proposal	This proposal aims to evaluate the response to treatment with intravenous and subcutaneous (20%) immunoglobulin in a series of patients with inflammatory idiopathic myopathies by using artificial intelligence	Predicting the clinical outcome

**Table 6:** Comparative studies in omics data.

Ref.	Year	Models	Model	Omics Data	Comparison Parameters
[97]	2022	SVM, RF, NN, and NB	RF	Single omics (RNA)	Immune checkpoint blockade in gastric cancer patients
[98]	2019	LSTM-VAE, DCEC, K-means, HC, PAM	DCEC	Multi-omics proteins and metabolites	Number of significantly enriched biological pathways by each clustering method

**Table 7:** Available omics databases.

Ref.	Dataset Name	Data Type	Number of Samples	Case Study	Source	Comment
[99]	LncRNADisease	lncRNA	200,000	-	http://www.rnanut.net/lncrnadisease/ (accessed on 23 December 2022)	A database of the collection of experimentally supported lncRNA disease associations
[100]	Comparative Toxicogenomics Database	Gene products and phenotypes	38 million toxicogenomic relationships	-	http://ctdbase.org/about/;jsessionid=E3F199EC890421874604E21270A16338 (accessed on 23 December 2022)	Chemical–gene/protein interactions and chemical–disease and gene–disease relationships
[101]	NONCODE	RNAs	548,640	-	http://www.noncode.org/ (accessed on 23 December 2022)	Collection and annotation of noncoding RNAs (ncRNAs), particularly lncRNAs
[102]	Lnc2Cancer	lncRNA	4989	165 types of human cancer	-	Comprehensive experimentally supported associations between lncRNAs and human cancers
[103]	MNDR v3.0	ncRNA	One million entries	-	http://www.rnadisease.org/ (accessed on 23 December 2022)	Updated the mammal ncRNA–disease repository for investigation of disease mechanisms and clinical treatment strategies
[34]	TCGA	Genomic, epigenomic, transcriptomic, and proteomic data	2.5 petabytes	33 cancer types	https://www.cancer.gov/about-nci/organization/ccg/research/structural-genomics/tcga (accessed on 23 December 2022) https://portal.gdc.cancer.gov/ (accessed on 23 December 2022)	A landmark cancer genomics program molecularly characterized more than 20,000 primary cancers and matched normal samples spanning 33 cancer types
[104]	Reactome	Molecular details of signal transduction, transport, DNA replication, metabolism, and other cellular processes	-	Cardiovascular disease	https://reactome.org/download-data (accessed on 23 December 2022)	Archive of biological processes and as a tool for discovering functional relationships in data, such as gene expression profiles or somatic mutation catalogs from tumor cells
http://asia.ensembl.org/Homo_sapiens/Info/Annotation (accessed on 23 December 2022)	Reactome (version 70)	Human protein-coding genes	10,867	-	http://asia.ensembl.org/Homo_sapiens/Info/Annotation (accessed on 23 December 2022)	Collection of gene models built from the gene-wise alignments of the human proteome and alignments of human cDNAs using the cDNA2genome model of exonerate
[105]	cBioPortal	Genomic data	More than 5000 tumor samples from 20 cancer studies	Cancer	http://www.cbioportal.org/ (accessed on 23 December 2022)	Pen-access resource for interactive exploration of multidimensional cancer genomics datasets
[106]	DisGeNET	Gene-disease associations (GDAs)	1,134,942	-	https://www.disgenet.org/ (accessed on 23 December 2022)	Collections of genes and variants associated with human diseases
https://www.ncbi.nlm.nih.gov/gds (accessed on 23 December 2022)	GEO	Gene expression	-	-	https://www.ncbi.nlm.nih.gov/gds (accessed on 23 December 2022)	Collection of curated gene expression datasets and original series and platform records in the Gene Expression Omnibus (GEO) repository
https://www.ncbi.nlm.nih.gov/ (accessed on 23 December 2022)	NCBI	Genomic data	-	-	https://www.ncbi.nlm.nih.gov/ (accessed on 23 December 2022)	Search engine for discovering different biomedical and genomic information
https://string-db.org/cgi/about (accessed on 23 December 2022)	STRING	Protein	24,584,628	-	https://string-db.org/cgi/about (accessed on 23 December 2022)	A database of known and predicted PPIs
https://www.ensembl.org/index.html?redirect=no (accessed on 23 December 2022)	Ensemble	Genomic data	974,444	-	https://www.ensembl.org/index.html?redirect=no (accessed on 23 December 2022)	Search engine for discovering different genomic information
[107]	OMIM	Disease–gene association	-	-	https://www.omim.org/ (accessed on 23 December 2022)	A resource of curated descriptions of human genes and phenotypes and the relationships between them
https://www.expasy.org/resources/uniprotkb-swiss-prot (accessed on 23 December 2022)	SWISS–PROT	Protein sequence	-	-	https://www.expasy.org/resources/uniprotkb-swiss-prot (accessed on 23 December 2022)	Contains protein descriptions, including function, domain structure, subcellular location, posttranslational modifications, and functionally characterized variants
https://www.ebi.ac.uk/ena/browser/home (accessed on 23 December 2022)	EMBI	Nucleic acid sequence	-	-	https://www.ebi.ac.uk/ena/browser/home (accessed on 23 December 2022)	A comprehensive record of the world’s nucleotide sequencing information, covering raw sequencing data, sequence assembly information, and functional annotation
https://www.ddbj.nig.ac.jp/index-e.html (accessed on 23 December 2022)	DDBJ	Nucleic acid sequence	-	-	https://www.ddbj.nig.ac.jp/index-e.html (accessed on 23 December 2022)	-
http://scop.mrc-lmb.cam.ac.uk/ (accessed on 23 December 2022)	SCOP	Protein structure classification	861,631	-	http://scop.mrc-lmb.cam.ac.uk/ (accessed on 23 December 2022)	A comprehensive description of the structural and evolutionary relationships between all proteins whose structure is known

## Data Availability

Not applicable.

## References

[1] Pan Y., Lei X., Zhang Y. (2022). Association predictions of genomics, proteinomics, transcriptomics, microbiome, metabolomics, pathomics, radiomics, drug, symptoms, environment factor, and disease networks: A comprehensive approach. Med. Res. Rev..

[2] Zhang Z., Zhao Y., Liao X., Shi W., Li K., Zou Q., Peng S. (2018). Deep learning in omics: A survey and guideline. Brief. Funct. Genom..

[3] Rong Z., Liu Z., Song J., Cao L., Yu Y., Qiu M., Hou Y. (2022). MCluster-VAEs: An end-to-end variational deep learning-based clustering method for subtype discovery using multi-omics data. Comput. Biol. Med..

[4] Mohammed M.A., Lakhan A., Abdulkareem K.H., Zapirain B.G. (2023). A hybrid cancer prediction based on multi-omics data and reinforcement learning state action reward state action (SARSA). Comput. Biol. Med..

[5] Verhaak R.G., Hoadley K.A., Purdom E., Wang V., Qi Y., Wilkerson M.D., Miller C.R., Ding L., Golub T., Mesirov J.P. (2010). Integrated genomic analysis identifies clinically relevant subtypes of glioblastoma characterized by abnormalities in PDGFRA, IDH1, EGFR, and NF1. Cancer Cell.

[6] Phillips H.S., Kharbanda S., Chen R., Forrest W.F., Soriano R.H., Wu T.D., Misra A., Nigro J.M., Colman H., Soroceanu L. (2006). Molecular subclasses of high-grade glioma predict prognosis, delineate a pattern of disease progression, and resemble stages in neurogenesis. Cancer Cell.

[7] Brennan C.W., Verhaak R.G., McKenna A., Campos B., Noushmehr H., Salama S.R., Zheng S., Chakravarty D., Sanborn J.Z., Berman S.H. (2013). The somatic genomic landscape of glioblastoma. Cell.

[8] Noushmehr H., Weisenberger D.J., Diefes K., Phillips H.S., Pujara K., Berman B.P., Pan F., Pelloski C.E., Sulman E.P., Bhat K.P. (2010). Identification of a CpG island methylator phenotype that defines a distinct subgroup of glioma. Cancer Cell.

[9] Sarra R.R., Dinar A.M., Mohammed M.A., Abdulkareem K.H. (2022). Enhanced Heart Disease Prediction Based on Machine Learning and &chi;2 Statistical Optimal Feature Selection Model. Designs.

[10] Mohammed M.A., Elhoseny M., Abdulkareem K.H., Mostafa S.A., Maashi. M.S. (2021). A Multi-agent Feature Selection and Hybrid Classification Model for Parkinson’s Disease Diagnosis. ACM Trans. Multimed. Comput. Commun. Appl..

[11] Abd Ghani M.K., Noma N.G., Mohammed M.A., Abdulkareem K.H., Garcia-Zapirain B., Maashi M.S., Mostafa S.A. (2021). Innovative Artificial Intelligence Approach for Hearing-Loss Symptoms Identification Model Using Machine Learning Techniques. Sustainability.

[12] Hameed Abdulkareem K., Awad Mutlag A., Musa Dinar A., Frnda J., Abed Mohammed M., Hasan Zayr F., Lakhan A., Kadry S., Ali Khattak H., Nedoma J. (2022). Smart Healthcare System for Severity Prediction and Critical Tasks Management of COVID-19 Patients in IoT-Fog Computing Environments. Comput. Intell. Neurosci..

[13] Abdulkareem K.H., Mostafa S.A., Al-Qudsy Z.N., Mohammed M.A., Al-Waisy A.S., Kadry S., Lee J., Nam Y. (2022). Automated System for Identifying COVID-19 Infections in Computed Tomography Images Using Deep Learning Models. J. Healthc. Eng..

[14] Abdulkareem K.H., Al-Mhiqani M.N., Dinar A.M., Mohammed M.A., Al-Imari M.J., Al-Waisy A.S., Alghawli A.S., Al-Qaness M.A.A. (2022). MEF: Multidimensional Examination Framework for Prioritization of COVID-19 Severe Patients and Promote Precision Medicine Based on Hybrid Multi-Criteria Decision-Making Approaches. Bioengineering.

[15] Zhu Y., Ouyang Z., Du H., Wang M., Wang J., Sun H., Kong L., Xu Q., Ma H., Sun Y. (2022). New opportunities and challenges of natural products research: When target identification meets single-cell multiomics. Acta Pharm. Sin. B.

[16] Liu B., Liu Y., Pan X., Li M., Yang S., Li S.C. (2019). DNA Methylation Markers for Pan-Cancer Prediction by Deep Learning. Genes.

[17] Pan X., Liu B., Wen X., Liu Y., Zhang X., Li S., Li S. (2019). D-GPM: A Deep Learning Method for Gene Promoter Methylation Inference. Genes.

[18] Singh R., Lanchantin J., Robins G., Qi Y. (2016). DeepChrome: Deep-learning for predicting gene expression from histone modifications. Bioinformatics.

[19] Xiong H.Y., Alipanahi B., Lee L.J., Bretschneider H., Merico D., Yuen R.K., Hua Y., Gueroussov S., Najafabadi H.S., Hughes T.R. (2015). RNA splicing. The human splicing code reveals new insights into the genetic determinants of disease. Science.

[20] Ashley E.A. (2016). Towards precision medicine. Nat. Rev. Genet..

[21] Chen R., Snyder M. (2013). Promise of personalized omics to precision medicine. Wiley Interdiscip. Reviews. Syst. Biol. Med..

[22] Martorell-Marugán J., Tabik S., Benhammou Y., del Val C., Zwir I., Herrera F., Carmona-Sáez P. (2019). Deep learning in omics data analysis and precision medicine. Computational Biology.

[23] Nicora G., Vitali F., Dagliati A., Geifman N., Bellazzi R. (2020). Integrated Multi-Omics Analyses in Oncology: A Review of Machine Learning Methods and Tools. Front. Oncol..

[24] Cristovao F., Cascianelli S., Canakoglu A., Carman M., Nanni L., Pinoli P., Masseroli M. (2022). Investigating Deep Learning Based Breast Cancer Subtyping Using Pan-Cancer and Multi-Omic Data. IEEE/ACM Trans. Comput. Biol. Bioinform..

[25] Tu W., Zhou S., Liu X., Guo X., Cai Z., Zhu E., Cheng J. Deep fusion clustering network. Proceedings of the AAAI Conference on Artificial Intelligence.

[26] Rakshit S., Saha I., Chakraborty S.S., Plewczyski D. Deep learning for integrated analysis of breast cancer subtype specific multi-omics data. Proceedings of the TENCON 2018-2018 IEEE Region 10 Conference.

[27] Young J.D., Cai C., Lu X. (2017). Unsupervised deep learning reveals prognostically relevant subtypes of glioblastoma. BMC Bioinform..

[28] Rhee S., Seo S., Kim S. (2017). Hybrid approach of relation network and localized graph convolutional filtering for breast cancer subtype classification. arXiv.

[29] Shuangshuang L., Lin Q., Yun T., Fenghui L. A Deep Learning Fusion Clustering framework for breast cancer subtypes identification by integrating multi-omics data. Proceedings of the 2020 5th International Conference on Mechanical, Control and Computer Engineering (ICMCCE).

[30] Viaud G., Mayilvahanan P., Cournède P.H. (2022). Representation Learning for the Clustering of Multi-Omics Data. IEEE/ACM Trans. Comput. Biol. Bioinform..

[31] Li B., Wang T., Nabavi S. Cancer molecular subtype classification by graph convolutional networks on multi-omics data. Proceedings of the 12th ACM Conference on Bioinformatics, Computational Biology, and Health Informatics.

[32] Strimbu K., Tavel J.A. (2010). What are biomarkers?. Curr. Opin. HIV AIDS.

[33] Lin Y., Qian F., Shen L., Chen F., Chen J., Shen B. (2019). Computer-aided biomarker discovery for precision medicine: Data resources, models and applications. Brief. Bioinform..

[34] Chang K., Creighton C.J., Davis C., Donehower L., Drummond J., Wheeler D., Ally A., Balasundaram M., Birol I., Butterfield Y.S.N. (2013). The Cancer Genome Atlas Pan-Cancer analysis project. Nat. Genet..

[35] Debnath M., Prasad G.B., Bisen P.S. (2010). Molecular Diagnostics: Promises and Possibilities.

[36] Shrivastava A.K., Singh H.V., Raizada A., Singh S.K. (2015). C-reactive protein, inflammation and coronary heart disease. Egypt. Heart J..

[37] Le N., Sund M., Vinci A. (2016). Prognostic and predictive markers in pancreatic adenocarcinoma. Dig. Liver Dis..

[38] Mandel S.A., Morelli M., Halperin I., Korczyn A.D. (2010). Biomarkers for prediction and targeted prevention of Alzheimer’s and Parkinson’s diseases: Evaluation of drug clinical efficacy. EPMA J..

[39] Reel P.S., Reel S., Pearson E., Trucco E., Jefferson E. (2021). Using machine learning approaches for multi-omics data analysis: A review. Biotechnol. Adv..

[40] Pham T.-H., Qiu Y., Zeng J., Xie L., Zhang P. (2021). A deep learning framework for high-throughput mechanism-driven phenotype compound screening and its application to COVID-19 drug repurposing. Nat. Mach. Intell..

[41] Azuaje F. (2017). Computational models for predicting drug responses in cancer research. Brief. Bioinform..

[42] Wang Y., Zhang Z., Chai H., Yang Y. Multi-omics Cancer Prognosis Analysis Based on Graph Convolution Network. Proceedings of the 2021 IEEE International Conference on Bioinformatics and Biomedicine (BIBM).

[43] Park C., Oh I., Choi J., Ko S., Ahn J. (2021). Improved Prediction of Cancer Outcome Using Graph-Embedded Generative Adversarial Networks. IEEE Access.

[44] Liu X., Xu X., Xu X., Li X., Xie G. Representation Learning for Multi-omics Data with Heterogeneous Gene Regulatory Network. Proceedings of the 2021 IEEE International Conference on Bioinformatics and Biomedicine (BIBM).

[45] Dutta P., Patra A.P., Saha S. (2022). DeePROG: Deep Attention-Based Model for Diseased Gene Prognosis by Fusing Multi-Omics Data. IEEE/ACM Trans. Comput. Biol. Bioinform..

[46] Peng C., Zheng Y., Huang D.S. (2020). Capsule Network Based Modeling of Multi-omics Data for Discovery of Breast Cancer-Related Genes. IEEE/ACM Trans. Comput. Biol. Bioinform..

[47] Daoud S., Mdhaffar A., Jmaiel M., Freisleben B. (2020). Q-Rank: Reinforcement Learning for Recommending Algorithms to Predict Drug Sensitivity to Cancer Therapy. IEEE J. Biomed. Health Inform..

[48] Nguyen T.H., Prifti E., Sokolovska N., Zucker J.D. Disease Prediction Using Synthetic Image Representations of Metagenomic Data and Convolutional Neural Networks. Proceedings of the 2019 IEEE-RIVF International Conference on Computing and Communication Technologies (RIVF).

[49] Matsubara T., Ochiai T., Hayashida M., Akutsu T., Nacher J.C. (2019). Convolutional neural network approach to lung cancer classification integrating protein interaction network and gene expression profiles. J. Bioinform. Comput. Biol..

[50] Sehanobish A., Ravindra N., van Dijk D. (2021). Gaining Insight into SARS-CoV-2 Infection and COVID-19 Severity Using Self-supervised Edge Features and Graph Neural Networks. Proc. AAAI Conf. Artif. Intell..

[51] Park C., Ha J., Park S. (2020). Prediction of Alzheimer’s disease based on deep neural network by integrating gene expression and DNA methylation dataset. Expert Syst. Appl..

[52] Kong Y., Yu T. (2020). forgeNet: A graph deep neural network model using tree-based ensemble classifiers for feature graph construction. Bioinformatics.

[53] Alzubaidi A., Tepper J., Lotfi A. (2020). A novel deep mining model for effective knowledge discovery from omics data. Artif. Intell. Med..

[54] Schulte-Sasse R., Budach S., Hnisz D., Marsico A. (2019). Graph convolutional networks improve the prediction of cancer driver genes. Proceedings of the International Conference on Artificial Neural Networks.

[55] Luo P., Li Y., Tian L.-P., Wu F.-X. (2019). Enhancing the prediction of disease–gene associations with multimodal deep learning. Bioinformatics.

[56] Bourgeais V., Zehraoui F., Hanczar B. (2022). GraphGONet: A self-explaining neural network encapsulating the Gene Ontology graph for phenotype prediction on gene expression. Bioinformatics.

[57] Lee T.-Y., Huang K.-Y., Chuang C.-H., Lee C.-Y., Chang T.-H. (2020). Incorporating deep learning and multi-omics autoencoding for analysis of lung adenocarcinoma prognostication. Comput. Biol. Chem..

[58] Li H., Sun Y., Hong H., Huang X., Tao H., Huang Q., Wang L., Xu K., Gan J., Chen H. (2022). Inferring transcription factor regulatory networks from single-cell ATAC-seq data based on graph neural networks. Nat. Mach. Intell..

[59] Hassanzadeh H.R., Wang M.D. (2021). An Integrated Deep Network for Cancer Survival Prediction Using Omics Data. Front. Big Data.

[60] Chai H., Zhang Z., Wang Y., Yang Y. (2021). Predicting bladder cancer prognosis by integrating multi-omics data through a transfer learning-based Cox proportional hazards network. CCF Trans. High Perform. Comput..

[61] Chai H., Zhou X., Zhang Z., Rao J., Zhao H., Yang Y. (2021). Integrating multi-omics data through deep learning for accurate cancer prognosis prediction. Comput. Biol. Med..

[62] Khoshghalbvash F., Gao J.X. Integrating Heterogeneous Datasets by Using Multimodal Deep Learning. Proceedings of the Communications, Signal Processing, and Systems.

[63] Kipf T.N., Welling M. (2016). Semi-supervised classification with graph convolutional networks. arXiv.

[64] Ma Y. (2022). DeepMNE: Deep Multi-Network Embedding for lncRNA-Disease Association Prediction. IEEE J. Biomed. Health Inform..

[65] Zhang Y., Wang Z., Liu Y., Lu L., Tan X., Zou Q. By hybrid neural networks for prediction and interpretation of transcription factor binding sites based on multi-omics. Proceedings of the 2021 IEEE International Conference on Bioinformatics and Biomedicine (BIBM).

[66] Li S., Han H., Sui D., Hao A., Qin H. A Novel Radiogenomics Framework for Genomic and Image Feature Correlation using Deep Learning. Proceedings of the 2018 IEEE International Conference on Bioinformatics and Biomedicine (BIBM).

[67] Xu F., Guo G., Zhu F., Tan X., Fan L. (2021). Protein deep profile and model predictions for identifying the causal genes of male infertility based on deep learning. Inf. Fusion.

[68] Mortezaei Z., Tavallaei M. (2021). Novel directions in data pre-processing and genome-wide association study (GWAS) methodologies to overcome ongoing challenges. Inform. Med. Unlocked.

[69] Hess M., Hackenberg M., Binder H. (2020). Exploring generative deep learning for omics data using log-linear models. Bioinform..

[70] Xiao Q., Dai J., Luo J., Fujita H. (2019). Multi-view manifold regularized learning-based method for prioritizing candidate disease miRNAs. Knowl. Based Syst..

[71] Nicholls H.L., John C.R., Watson D.S., Munroe P.B., Barnes M.R., Cabrera C.P. (2020). Reaching the End-Game for GWAS: Machine Learning Approaches for the Prioritization of Complex Disease Loci. Front. Genet..

[72] Fu Y., Xu J., Tang Z., Wang L., Yin D., Fan Y., Zhang D., Deng F., Zhang Y., Zhang H. (2020). A gene prioritization method based on a swine multi-omics knowledgebase and a deep learning model. Commun. Biol..

[73] Ji Y., Lotfollahi M., Wolf F.A., Theis F.J. (2021). Machine learning for perturbational single-cell omics. Cell Syst..

[74] Mahmud M., Kaiser M.S., McGinnity T.M., Hussain A. (2021). Deep Learning in Mining Biological Data. Cogn. Comput..

[75] Albaradei S., Thafar M., Alsaedi A., Van Neste C., Gojobori T., Essack M., Gao X. (2021). Machine learning and deep learning methods that use omics data for metastasis prediction. Comput. Struct. Biotechnol. J..

[76] Lin E., Lin C.H., Lane H.Y. (2021). Deep Learning with Neuroimaging and Genomics in Alzheimer’s Disease. Int. J. Mol. Sci..

[77] Treppner M., Binder H., Hess M. (2022). Interpretable generative deep learning: An illustration with single cell gene expression data. Hum. Genet..

[78] Serra A., Fratello M., Cattelani L., Liampa I., Melagraki G., Kohonen P., Nymark P., Federico A., Kinaret P.A., Jagiello K. (2020). Transcriptomics in Toxicogenomics, Part III: Data Modelling for Risk Assessment. Nanomaterials.

[79] Mendez K.M., Broadhurst D.I., Reinke S.N. (2019). The application of artificial neural networks in metabolomics: A historical perspective. Metabolomics.

[80] Aghdam S.A., Brown A.M.V. (2021). Deep learning approaches for natural product discovery from plant endophytic microbiomes. Environ. Microbiome.

[81] Pomyen Y., Wanichthanarak K., Poungsombat P., Fahrmann J., Grapov D., Khoomrung S. (2020). Deep metabolome: Applications of deep learning in metabolomics. Comput. Struct Biotechnol. J..

[82] Xu F., Wang S., Dai X., Mundra P.A., Zheng J. (2021). Ensemble learning models that predict surface protein abundance from single-cell multimodal omics data. Methods.

[83] Wang X., Dong Y., Zheng Y., Chen Y. (2021). Multiomics metabolic and epigenetics regulatory network in cancer: A systems biology perspective. J. Genet. Genom. Yi Chuan Xue Bao.

[84] Schneider L., Laiouar-Pedari S., Kuntz S., Krieghoff-Henning E., Hekler A., Kather J.N., Gaiser T., Fröhling S., Brinker T.J. (2022). Integration of deep learning-based image analysis and genomic data in cancer pathology: A systematic review. Eur. J. Cancer.

[85] Eicher T., Kinnebrew G., Patt A., Spencer K., Ying K., Ma Q., Machiraju R., Mathé A.E.A. (2020). Metabolomics and Multi-Omics Integration: A Survey of Computational Methods and Resources. Metabolites.

[86] Wang X., Li B.B. (2021). Deep Learning in Head and Neck Tumor Multiomics Diagnosis and Analysis: Review of the Literature. Front. Genet..

[87] Tufail A.B., Ma Y.-K., Kaabar M.K.A., Martínez F., Junejo A.R., Ullah I., Khan R. (2021). Deep Learning in Cancer Diagnosis and Prognosis Prediction: A Minireview on Challenges, Recent Trends, and Future Directions. Comput. Math. Methods Med..

[88] Termine A., Fabrizio C., Strafella C., Caputo V., Petrosini L., Caltagirone C., Giardina E., Cascella R. (2021). Multi-Layer Picture of Neurodegenerative Diseases: Lessons from the Use of Big Data through Artificial Intelligence. J. Pers. Med..

[89] Krassowski M., Das V., Sahu S.K., Misra B.B. (2020). State of the Field in Multi-Omics Research: From Computational Needs to Data Mining and Sharing. Front. Genet..

[90] Alqahtani A. (2022). Application of Artificial Intelligence in Discovery and Development of Anticancer and Antidiabetic Therapeutic Agents. Evid. Based Complement. Altern. Med..

[91] Song M., Greenbaum J., Luttrell J.t., Zhou W., Wu C., Shen H., Gong P., Zhang C., Deng H.W. (2020). A Review of Integrative Imputation for Multi-Omics Datasets. Front. Genet..

[92] Azuaje F. (2019). Artificial intelligence for precision oncology: Beyond patient stratification. Npj Precis. Oncol..

[93] Holzinger A., Haibe-Kains B., Jurisica I. (2019). Why imaging data alone is not enough: AI-based integration of imaging, omics, and clinical data. Eur. J. Nucl. Med. Mol. Imaging.

[94] Dozmorov M.G. (2019). Disease classification: From phenotypic similarity to integrative genomics and beyond. Brief. Bioinform..

[95] Castañé H., Baiges-Gaya G., Hernández-Aguilera A., Rodríguez-Tomàs E., Fernández-Arroyo S., Herrero P., Delpino-Rius A., Canela N., Menendez J.A., Camps J. (2021). Coupling Machine Learning and Lipidomics as a Tool to Investigate Metabolic Dysfunction-Associated Fatty Liver Disease. A General Overview. Biomolecules.

[96] Danieli M.G., Tonacci A., Paladini A., Longhi E., Moroncini G., Allegra A., Sansone F., Gangemi S. (2022). A machine learning analysis to predict the response to intravenous and subcutaneous immunoglobulin in inflammatory myopathies. A proposal for a future multi-omics approach in autoimmune diseases. Autoimmun. Rev..

[97] Sung J.Y., Cheong J.H. (2022). Machine Learning Predictor of Immune Checkpoint Blockade Response in Gastric Cancer. Cancers.

[98] Chung N.C., Mirza B., Choi H., Wang J., Wang D., Ping P., Wang W. (2019). Unsupervised classification of multi-omics data during cardiac remodeling using deep learning. Methods.

[99] Bao Z., Yang Z., Huang Z., Zhou Y., Cui Q., Dong D. (2019). LncRNADisease 2.0: An updated database of long non-coding RNA-associated diseases. Nucleic Acids Res..

[100] Davis A.P., Grondin C.J., Johnson R.J., Sciaky D., McMorran R., Wiegers J., Wiegers T.C., Mattingly C.J. (2019). The Comparative Toxicogenomics Database: Update 2019. Nucleic Acids Res..

[101] Fang S., Zhang L., Guo J., Niu Y., Wu Y., Li H., Zhao L., Li X., Teng X., Sun X. (2018). NONCODEV5: A comprehensive annotation database for long non-coding RNAs. Nucleic Acids Res..

[102] Gao Y., Wang P., Wang Y., Ma X., Zhi H., Zhou D., Li X., Fang Y., Shen W., Xu Y. (2019). Lnc2Cancer v2.0: Updated database of experimentally supported long non-coding RNAs in human cancers. Nucleic Acids Res..

[103] Ning L., Cui T., Zheng B., Wang N., Luo J., Yang B., Du M., Cheng J., Dou Y., Wang D. (2021). MNDR v3.0: Mammal ncRNA-disease repository with increased coverage and annotation. Nucleic Acids Res..

[104] Fabregat A., Jupe S., Matthews L., Sidiropoulos K., Gillespie M., Garapati P., Haw R., Jassal B., Korninger F., May B. (2018). The Reactome Pathway Knowledgebase. Nucleic Acids Res..

[105] Cerami E., Gao J., Dogrusoz U., Gross B.E., Sumer S.O., Aksoy B.A., Jacobsen A., Byrne C.J., Heuer M.L., Larsson E. (2012). The cBio cancer genomics portal: An open platform for exploring multidimensional cancer genomics data. Cancer Discov..

[106] Piñero J., Bravo À., Queralt-Rosinach N., Gutiérrez-Sacristán A., Deu-Pons J., Centeno E., García-García J., Sanz F., Furlong L.I. (2017). DisGeNET: A comprehensive platform integrating information on human disease-associated genes and variants. Nucleic Acids Res..

[107] Amberger J.S., Bocchini C.A., Schiettecatte F., Scott A.F., Hamosh A. (2015). OMIM.org: Online Mendelian Inheritance in Man (OMIM®), an online catalog of human genes and genetic disorders. Nucleic Acids Res..

[108] Spencer M., Eickholt J., Cheng J. (2015). A Deep Learning Network Approach to ab initio Protein Secondary Structure Prediction. IEEE/ACM Trans. Comput. Biol. Bioinform..

[109] Date Y., Kikuchi J. (2018). Application of a Deep Neural Network to Metabolomics Studies and Its Performance in Determining Important Variables. Anal. Chem..

[110] Wu Z., Pan S., Chen F., Long G., Zhang C., Yu P.S. (2021). A Comprehensive Survey on Graph Neural Networks. IEEE Trans Neural Netw Learn. Syst.

[111] Defferrard M., Bresson X., Vandergheynst P. (2016). Convolutional neural networks on graphs with fast localized spectral filtering. Adv. Neural Inf. Process. Syst..

[112] Cai Z., Xu D., Zhang Q., Zhang J., Ngai S.M., Shao J. (2015). Classification of lung cancer using ensemble-based feature selection and machine learning methods. Mol. Biosyst..

[113] Breiman L. (2001). Random Forests. Mach. Learn..

[114] Perou C.M., Sørlie T., Eisen M.B., van de Rijn M., Jeffrey S.S., Rees C.A., Pollack J.R., Ross D.T., Johnsen H., Akslen L.A. (2000). Molecular portraits of human breast tumours. Nature.

[115] Nguyen N.D., Jin T., Wang D. (2021). Varmole: A biologically drop-connect deep neural network model for prioritizing disease risk variants and genes. Bioinformatics.

[116] Tran N., Gao J. (2020). Network Representation of Large-Scale Heterogeneous RNA Sequences with Integration of Diverse Multi-omics, Interactions, and Annotations Data. Pacific Symposium on Biocomputing. Pac. Symp. Biocomput..

[117] Luo P., Tian L.-P., Chen B., Xiao Q., Wu F.-X. (2018). Predicting Gene-Disease Associations with Manifold Learning. Proceedings of the International Symposium on Bioinformatics Research and Applications.

[118] Hutson M. (2018). Artificial intelligence faces reproducibility crisis. Science.

[119] Vaswani A., Bengio S., Brevdo E., Chollet F., Gomez A.N., Gouws S., Jones L., Kaiser Ł., Kalchbrenner N., Parmar N. (2018). Tensor2tensor for neural machine translation. arXiv.

[120] Jo T., Nho K., Saykin A.J. (2019). Deep Learning in Alzheimer’s Disease: Diagnostic Classification and Prognostic Prediction Using Neuroimaging Data. Front. Aging Neurosci..

[121] Brodersen K.H., Ong C.S., Stephan K.E., Buhmann J.M. The Balanced Accuracy and Its Posterior Distribution. Proceedings of the 2010 20th International Conference on Pattern Recognition.

[122] Burrell R.A., McGranahan N., Bartek J., Swanton C. (2013). The causes and consequences of genetic heterogeneity in cancer evolution. Nature.

[123] Jiang X., Zhao J., Qian W., Song W., Lin G.N. (2020). A Generative Adversarial Network Model for Disease Gene Prediction With RNA-seq Data. IEEE Access.

[124] Kaur P., Singh A., Chana I. (2021). Computational Techniques and Tools for Omics Data Analysis: State-of-the-Art, Challenges, and Future Directions. Arch. Comput. Methods Eng..

[125] Miotto R., Wang F., Wang S., Jiang X., Dudley J.T. (2018). Deep learning for healthcare: Review, opportunities and challenges. Brief. Bioinform..

[126] Picard M., Scott-Boyer M.P., Bodein A., Périn O., Droit A. (2021). Integration strategies of multi-omics data for machine learning analysis. Comput. Struct. Biotechnol. J..

[127] Mirza B., Wang W., Wang J., Choi H., Chung N.C., Ping P. (2019). Machine Learning and Integrative Analysis of Biomedical Big Data. Genes.

[128] Gunning D., Stefik M., Choi J., Miller T., Stumpf S., Yang G.Z. (2019). XAI-Explainable artificial intelligence. Sci. Robot..

